# Safety Net Programs as Primary Prevention Against Adverse Childhood Experiences (ACEs) in the United States: Natural Experiments with Temporary Assistance for Needy Families (TANF) and Supplemental Nutrition Assistance Program (SNAP)

**DOI:** 10.3390/ijerph22111750

**Published:** 2025-11-19

**Authors:** Tasfia Jahangir, Briana Woods-Jaeger, Kelli A. Komro, Melvin D. Livingston

**Affiliations:** Department of Behavioral, Social, and Health Education Sciences, Rollins School of Public Health, Emory University, Atlanta, GA 30322, USA

**Keywords:** adverse childhood experiences, welfare, social policy, primary prevention, child health, child maltreatment

## Abstract

We examine access to U.S. welfare programs—Temporary Assistance for Needy Families (TANF) and Supplemental Nutrition Assistance Program (SNAP)—as primary prevention strategies against adverse childhood experiences (ACEs). Using the University of Kentucky’s National Welfare Data and National Survey of Children’s Health (2016–2022), we estimate two-way fixed effects models linking state-level access rates to child-level ACE incidence. TANF access predicts reduced parental mental illness (fully adjusted β = −5.40, 95% CI: −8.80, −2.00), and parental incarceration in the model adjusted for state-level factors (β = −4.44, 95% CI: −8.84, −0.05), though the latter attenuates with child-level covariate adjustment. Unexpectedly, SNAP access correlates with slight increases in neighborhood violence exposure (fully adjusted β = 0.95, 95% CI: 0.39, 1.51) and parental substance use (crude β = 0.48, 95% CI: 0.04, 0.93) in crude models. Robustness checks show greater TANF access is associated with fewer total ACEs (β = −0.27, 95% CI: −0.46, −0.07). Results suggest that welfare programs hinge on broader social contexts; TANF access appears protective, while SNAP findings diverge from prior research, likely reflecting measurement or contextual limitations that merit careful further investigation, rather than overinterpretations of program harm.

## 1. Introduction

Poverty and childhood adversity are inextricably linked [1,2], with negative lifelong health consequences [3,4]. While adverse childhood experiences, or ACEs—defined as child abuse, neglect, and household or community instability—may affect children across the income gradient, they are more likely to compound in marginalized communities rendered vulnerable by poverty, limited public resources, and other drivers of systemic inequity [5,6]. This relationship exists across multiple levels of the social ecology (e.g., individual, family, neighborhood) [5,7,8], and is corroborated by research that examines other measures of child maltreatment instead of the ACE index [9,10,11,12,13,14].

A 2019 study using data from the National Survey of Children’s Health (NSCH) linked a high prevalence of ACEs to economic hardship, calling for family economic supports to strengthen financial security and, in turn, prevent children’s exposure to violence or neglect in the home [15]. Policies such as Temporary Assistance for Needy Families (TANF) and Supplemental Nutrition Assistance Program (SNAP), which supplement basic needs in low-income families, create potential avenues for intervention. TANF primarily functions as a cash assistance or household income support program, delivered through electronic benefit cards that function as debit cards and can be used for most basic goods and services (with some restrictions), or direct deposit [16]. Notably, states are afforded considerable discretion to redirect federal TANF dollars to other uses. SNAP, on the other hand, is a near-cash supplement for purchasing food products [17]. As the two largest federally funded means-tested programs in the American safety net [17], TANF and SNAP are pertinent to the study of ACEs. Indeed, the statutory purpose of TANF is “to provide assistance to needy families so that children can be cared for at home” [18], whereas children comprise 35% of the SNAP caseload [17]. As such, both programs are theoretically well-situated to address the risks associated with childhood poverty.

However, the current accessibility and design of both TANF and SNAP were shaped under the 1996 Personal Responsibility and Work Opportunity Reconciliation Act (PRWORA), although in different ways. PRWORA is a centerpiece of the Clinton administration’s campaign to “end welfare as we know it” and consolidate the broader neoliberal turn in U.S. social policy. Aid to Families with Dependent Children (AFDC), created in 1935, had provided an open-ended entitlement to cash assistance to families in poverty, under which states were entitled to unlimited federal funds for reimbursement of benefit payments at “matching” rates inversely related to state per capita income, ensuring that benefits expanded to meet need [18]. PRWORA dismantled AFDC, replacing it with TANF as a capped block grant that imposed work requirements, lifetime limits, and state discretion to redirect funds away from direct aid [19,20]. This restructuring, animated by racialized and gendered narratives of dependency and the politics of austerity, transformed cash assistance from a redistributive guarantee for eligible families into a conditional program, shrinking caseloads and eroding benefits [19,20]. As a result, TANF’s reach has sharply declined: in 2020, only 21 out of every 100 families in poverty received TANF benefits, compared to 68 out of 100 in 1996 through AFDC [20]. SNAP, which had existed since the 1960s as the Food Stamp Program, was not eliminated by PRWORA, but the law imposed new restrictions, including a five-year bar for many lawful immigrants, alongside expanded work requirements [21]. While SNAP was later renamed in 2008 and has remained a federal entitlement program with broad reach, TANF has become increasingly constrained and state-driven [20,21,22]. These divergent trajectories (one entitlement weakened but preserved, the other transformed into a strict block grant) may shape children’s exposure to adversity through different mechanisms.

In comparative terms, while TANF and SNAP are distinctive to the U.S. policy landscape, they align with broader categories of global social protection programs. Using Mays et al.’s (2010) typology of public health delivery systems, TANF most closely resembles redistributive income-support mechanisms embedded in social service systems [23]. Yet, unlike open-ended entitlements that expand to meet need, TANF’s block grant structure caps funding, imposes conditions, and allows states to restrict or divert aid [20], making it far less redistributive than traditional cash transfers. On the other hand, SNAP reflects a nutrition focused assurance activity blending oversight with private market distribution [23]. Comparable programs exist worldwide: cash transfers in Latin America, Africa and Asia aim for poverty reduction and improved child health, while food subsidy and voucher systems in the European Union and the World Food Programme stabilize nutrition and household security [24,25,26,27,28,29,30]. Thus, TANF and SNAP share features with global cash transfer and food supplementation programs, though in the U.S. they have taken on a neoliberal form shaped by conditionality, restricted benefits, and individualized responsibility, even as they remain formally oriented toward poverty reduction and well-being. These features raise important questions about the scale and potential of TANF and SNAP as primary prevention strategies for ACEs—questions that remain largely underexplored.

The extant literature has primarily focused on the impact of TANF and SNAP policies using limited measures of child maltreatment [10,31] and interpersonal violence [32,33]. Less is known about the extent to which these policies may prevent a more comprehensive range of ACEs. On one hand, by providing cash assistance, child care support, and monthly funds to purchase groceries, policies such as SNAP and TANF may hold promise in preventing ACE exposure by intervening on two salient risk factors for child maltreatment and neglect: financial and food insecurity [1,6,7,9,10,11,12,13,14,31,34,35,36,37,38,39,40]. On the other hand, there is some qualitative evidence from families and service providers that these programs may not realize their full potential due to restrictive design features (e.g., work requirements, sanctions for “noncompliance”, capped or diverted funding, time limits) and austerity measures (e.g., budget cuts, caseload reduction incentives, and stagnating investments in federal and state programs) [5,41,42]. Such a counterbalance is best conceptualized by the Ecological-Transactional Model of Child Maltreatment. This framework posits that a balance of *compensatory factors* that decrease the probability of child maltreatment (e.g., availability of social support and economic resources), and *potentiating factors* that increase the probability of child maltreatment (e.g., constrained, insufficient, or unavailable support systems) dynamically influences the environmental conditions for child development. When potentiating factors outweigh compensatory factors, child maltreatment occurs [43,44]. We apply this framework to examine whether TANF and SNAP access, as economic supports, may shape this balance by addressing poverty as a risk factor for ACEs.

Furthermore, a systematic review of 25 systematic reviews revealed that most interventions for ACEs are predominantly secondary or tertiary in nature—that is, they are aimed at intervening after the exposure to adversity has already occurred (secondary) or mitigating its long-term consequences (tertiary). As such, they predominantly focus on psychological and behavioral mechanisms (e.g., cognitive behavioral therapy, parent training). Related efforts at other ecological levels include home visiting programs [45]; school-based social-emotional learning curricula [46,47]; and community coalition models such as Communities That Care [48]; and trauma-informed care approaches integrated into healthcare and school systems [49,50,51]. While these strategies are aimed at addressing individual, family, and community-level processes, there remains limited empirical evidence on the role of primary prevention strategies targeting the upstream social factors that *initiate* ACEs [52]. To advance the literature and intervention development, we examine welfare participation as a primary prevention strategy against ACEs. Specifically, we focus on two research questions:

1. Between 2016 and 2022, how were changes in state-level SNAP access associated with ACEs in families likely to benefit from welfare?
2. Between 2016 and 2022, how were changes in state-level TANF access associated with ACEs in families likely to benefit from welfare?

## 2. Materials and Methods

We analyze state-level repeated cross-sectional data to determine the relationship between changes in welfare access rates and ACE incidence in the United States. To minimize instrumentation bias due to changes in outcome data collection (i.e., from phone-based to electronic or mailed surveys), we restrict the sample period to fiscal years 2016–2022.

### 2.1. Ethics

All data are deidentified and derived from public sources. This study did not require approval from an institutional review board. Per the Emory University Institutional Review Board’s Non-Human Subjects Research Determination Form [53], this study was neither deemed research with “human subjects” nor a “clinical investigation”.

### 2.2. Data Sources and Measures

Our selection of exposures, outcomes and covariates was informed by the Ecological-Transactional Model of Child Maltreatment, which emphasizes how compensatory and potentiating factors interact to shape children’s risk of adversity [43,44]. TANF and SNAP access rates were hypothesized as compensatory factors: structural resources that could offset risks associated with poverty and food insecurity. We operationalized outcomes as ACEs, which represent widely used indicators of child maltreatment and exposure to instability. State-level covariates were included to capture broader ecological conditions that may function as either compensatory (e.g., higher minimum wages, robust school meal participation) or potentiating (e.g., high poverty rates, unemployment) factors related to ACE risk [54,55,56,57,58,59,60,61,62,63]. Similarly, child-level covariates (e.g., race/ethnicity, age, sex) were incorporated to account for patterned inequities, recognizing that these factors amplify or buffer adversity depending on social and structural context [17,19,64,65,66,67,68,69,70,71,72,73,74]. Of note, race and ethnicity are reported separately in the NSCH. Respondents identifying as Hispanic or Latino may be of any race.

#### 2.2.1. Welfare Access

Exposures of interest include state-level TANF and SNAP access rates, calculated by dividing the total number of people receiving TANF or SNAP by the total number of people in poverty. These counts are from fiscal years 2016 to 2022. We obtained these counts from state-level panel data from the University of Kentucky’s National Welfare Data, which is annually updated to reflect states’ economic conditions as they relate to labor participation, wages, welfare eligibility and caseloads, poverty, and other factors [75]. This data is compiled from multiple sources, including U.S. Department of Health and Human Services (HHS), U.S. Department of Agriculture, Food, and Nutrition Service (USDA), and the U.S. Census Bureau.

#### 2.2.2. Adverse Childhood Experiences (ACEs)

Outcomes of interest include a suite of ACE variables at the child level. We obtained this data from the NSCH, the largest annual national and state-level survey of the health needs of children aged 0–17. Parents or guardians complete the NSCH electronically or by mail, reporting on healthcare, family dynamics, parental health, neighborhood conditions, and school characteristics. All outcome variables were from calendar years 2016 to 2022.

For the individual ACEs, the first outcome variable was derived from the question, *“Since this child was born, how often has it been very hard to get by on your family’s income—hard to cover the basics like food or housing?”* This variable was included to elucidate any first-order associations of welfare participation on basic needs. To maintain consistency with the other ACE variables, we binarized the Likert scale response options by collapsing *Never* and *Rarely* into one category (“No”) and *Somewhat Often* and *Very Often* into another (“Yes”).

The next set of individual ACE variables was derived from an index with the question stem, *“To the best of your knowledge, has this child EVER experienced any of the following?”*, followed by eight items: *“Parent or guardian divorced or separated”, “Parent or guardian died”, “Parent or guardian served time in jail”, “Saw or heard parents or adults slap, hit, kick, punch one another in the home”, “Was a victim of violence or witnessed violence in the neighborhood”, “Lived with anyone who was mentally ill, suicidal, or severely depressed”, “Lived with anyone who had a problem with alcohol or drugs”, “Treated or judged unfairly because of his or her race or ethnic group”*. Response options for these questions were binary (“Yes”/“No”). These variables were included to evaluate any second-order associations of welfare participation on ACEs.

#### 2.2.3. Covariates

We include state and year fixed effects to control for all time-invariant covariates across states, as well as any changes common to all states over time. This means that any stable state characteristics (e.g., political culture, demographic composition, etc.) are absorbed by state fixed effects, eliminating their potential to bias estimates. Similarly, year fixed effects adjust for national shocks or secular trends (e.g., changes in federal policies, nationwide economic cycles and events such as the COVID-19 pandemic) that could simultaneously shape welfare access and ACE incidence across all states. Together, these fixed effects strengthen causal inference by ensuring that estimates reflect only within-state changes in TANF or SNAP access over time, rather than stable differences between states or nationwide shifts affecting all states simultaneously.

Thus, our adjusted models only required the addition of state-specific time-varying covariates. We selected covariates based on theoretical relevance, prior empirical evidence, and data availability. State-level covariates include unemployment rate, state minimum wage, participation in the National School Lunch Program, participation in the School Breakfast Program, and poverty rate. State-level unemployment rates and minimum wages capture labor market conditions that may shape both welfare participation and ACE risk [54,55,56,57,58,59,60,61,62]. Children in SNAP- and TANF-participating households are categorically eligible for free school meals [76], making school meal program participation a potentially confounding nutritional safety net. State poverty rates index for broader structural disadvantage [63]. In models where TANF access rate was the exposure, SNAP access rate was treated as a covariate, and vice versa, to reduce confounding from program overlap. At the child-level, race, ethnicity, age, and sex were included due to well-documented disparities in both welfare access and ACE prevalence [17,19,64,65,66,67,68,69,70,71,72,73,74]. We were not able to include additional state-level child welfare factors because comparable data were not consistently available across all states and years. Given these constraints, we prioritized a parsimonious set of covariates most likely to confound the welfare-ACE relationship while ensuring data completeness across the analytic period. We derived state-level covariates from UKCPR’s National Welfare Data, which compiled this information from the Bureau of Labor Statistics (BLS), Wage and Hour Division (WHD), USDA, U.S. Census, HHS, and Tax Policy Center. We derived child-level variables from the NSCH.

### 2.3. Statistical Analysis

We calculated descriptive statistics for all variables of interest. We then utilized two-way fixed effects estimation with state and year fixed effects. Assuming no effect heterogeneity, state fixed effects accounted for time-invariant differences across states, and year fixed effects accounted any changes over time occurring in all states that may confound the relationship between welfare participation and ACEs. Based on data availability, and to attempt correct temporal ordering of the hypothesized association between TANF or SNAP access and subsequent ACE incidence, we relied on fiscal year program data for the exposures, and calendar year survey data for the outcome, building in an approximate six-month lag between measurement windows (for example, a fiscal policy in September 2017 predicts an outcome in March 2018). This alignment is aimed at strengthening the directionality of the associations by capturing program participation rates that precede ACE reports. We then modeled the relationship between changes in ACE incidence and changes in TANF and SNAP access in three stages: (1) first, with only state and year fixed effects, (2) second, the initial model plus state-level covariates, and (3) finally, a fully adjusted model with both state- and child-level covariates. To focus our analyses on families most likely to be welfare-eligible, we restricted the sample to families that reported *“less than high school”* or *“high school or GED”* as the highest level of parental education in the household; there is precedence for relying on caregiver education as a proxy for welfare eligibility in the social policy literature [77,78,79,80]. All analyses accounted for the complex survey design in the NSCH. We use listwise analysis and did not perform further imputation, relying on NSCH guidance and weighting adjustments for nonresponse [81]. For ease of interpretation, all models are expressed in a percentage point.

As a robustness check, we also conducted supplementary analysis where we examine the association between SNAP and TANF access on the count of ACEs. We constructed a combined ACE score by summing the total number of ACEs each child experienced. This continuous measure captures cumulative exposure to adversity and provides a comprehensive indicator of overall ACE burden. Linear models equivalent to those previously described were used to estimate these associations.

## 3. Results

Descriptive statistics for all outcome variables and covariates are reported in Table 1. The full sample comprised 42,591 child-year observations across 50 states and the District of Columbia from 2016 to 2022. Model-specific analytic sample sizes ranged from 40,418 to 41,520 due to item nonresponse (~5% missingness overall). The average child was 9.2 years old; 52% were male, 43% Hispanic, 63% White, and 17% Black. ACE prevalence included 25% reporting difficulty covering basic needs, 30% parental divorce or separation, 12% parental incarceration, and 10% living with someone with an alcohol or drug problem. Other ACEs ranged from 4% (parent died or treated unfairly due to race) to 8% (living with someone mentally ill). At the state level, mean unemployment was 5.1%, minimum wage $8.82, poverty rate 12.0%, TANF participation 8.6%, and the SNAP gap 107 per 100 people in poverty.

Findings from the two-way fixed effects models are reported in Table 2. A 10-percentage-point increase in state-level TANF access among people in poverty was associated with a 4.4-percentage-point decrease in the probability that a child lived with a parent who had been incarcerated (β = −4.44, 95% CI: −8.84, −0.05) in models adjusted for state-level time-varying covariates. This association was attenuated and no longer statistically significant when models also accounted for child-level covariates (β = −3.90, 95% CI: −8.20, 0.41). Similarly, greater TANF access was associated with a 3.2-percentage-point lower probability of living with a mentally ill adult in the crude model (β = −3.20, 95% CI: −6.17, −0.23). In models adjusted for state covariates, greater TANF access was associated with a 5.7-percentage-point lower probability of living with a mentally ill adult (β = −5.67, 95% CI: −9.09, −2.26), and similar results were found for the fully adjusted model (β = −5.40, 95% CI: −8.80, −2.00). In contrast, increases in SNAP access had limited but statistically significant increases in two adverse exposures. A 10-percentage-point increase in SNAP access corresponded to a 0.63-percentage-point higher probability of exposure to neighborhood violence in the crude model (β = 0.63, 95% CI: 0.25, 1.01), a 0.99-point increase in the state-adjusted model (β = 0.99, 95% CI: 0.42, 1.57), and a 0.95-point increase in the fully adjusted model (β = 0.95, 95% CI: 0.39, 1.51). SNAP access was also positively associated with the probability of living with someone who had an alcohol or drug problem (β = 0.48, 95% CI: 0.04, 0.93) in the crude model only. No other statistically significant associations between state-level welfare access and individual ACEs were observed.

Robustness checks on the total count of ACEs found a similar pattern of results. There were small but statistically insignificant associations between SNAP access and the count of ACEs. A 10-percentage-point increase in state-level TANF access among people in poverty was associated with a decrease of −0.3 ACEs per child (β = −0.27, 95% CI: −0.46, −0.07).

## 4. Discussion

This study evaluates whether changes in the access to safety net programs, such as Temporary Assistance for Needy Families (TANF) and Supplemental Nutrition Assistance Program (SNAP), can reduce ACE exposure. We find that TANF access is protective for parental incarceration in our model accounting for state-level time-varying covariates, but not child-level covariates. TANF access is also protective for exposure to parental mental illness across all crude and adjusted models. Unexpectedly, SNAP access was associated with a slight increase in neighborhood violence exposure across crude and adjusted models, and exposure to parental substance use in the crude model. We found no associations between welfare access and individual ACEs. Our findings suggest that TANF access may operate as a compensatory factor within the Ecological-Transactional Model [43,44], buffering families against adversities such as parental mental illness and incarceration. The absence of protective associations for SNAP may reflect potentiating contextual factors, such as administrative burden and restricted eligibility that blunt its protective potential. Situating these results within this framework highlights how safety net programs can relate to exposure to ACEs, depending on their design, accessibility, and social context. The remainder of this section expands on these mechanisms in greater detail.

The protective relationship of TANF access on exposure to parental mental illness is well-aligned with prior research establishing welfare accessibility as a determinant of mental health [77,82,83,84,85]. While the present study measures TANF access as the proportion of individuals in poverty receiving welfare in a state, prior studies have examined other aspects of TANF accessibility, such as conditionality (i.e., sanctions, work requirements, welfare-to-work policies) [82,85], and the program’s adaptiveness to emergent crises such as COVID-19 (i.e., emergency cash provisions for those not already receiving TANF, waiving work requirements or sanctions, and automatic recertification for benefits) [77]. Relatedly, international studies involving middle- and high-income countries have also yielded similar findings, examining welfare accessibility in terms offering welfare advice in healthcare settings [84], and through various welfare expenditure systems and regimes (e.g., liberal, conservative, social democratic models) [83]. The consistency of these findings corroborates the current study’s conclusion that welfare access may play a protective role in mitigating parental mental health outcomes.

Our results on the protective relationship of TANF access on parental incarceration are largely consistent with prior work finding that economic support minimizes incarceration risk, particularly as it relates to recidivism and crime rates [86,87,88,89,90,91,92,93,94,95,96], with two exceptions [97,98]. However, this protective association of TANF on parental incarceration is only observed in the present study in a model that solely adjusts for state-level covariates. Once child-level covariates, such as race, ethnicity, sex, and age are introduced, the association between TANF access and parental incarceration attenuates. This suggests that the protective association of TANF access may not be robust enough to counteract the additional structural factors driving the disparities in incarceration risk across demographic groups. An example of such a factor may be the ban on accessing TANF for people with a drug felony conviction, which may disproportionally impact Black and Latino communities [99,100]; though Black and White Americans use illicit drugs at roughly the same rates, Black Americans make up 25% of drug arrests while comprising only 14% of the population [101,102,103,104]. Additionally, because incarceration is disproportionately experienced by men [105] while TANF receipt is largely administered through women [64], parental incarceration and welfare access are both gendered processes. However, it remains ambiguous how or why the child-level variables (i.e., gender, age, and race/ethnicity) help explain the protective relationship between TANF access and parental incarceration; further research is needed to understand these dynamics. Despite the attenuation, this relationship remains an important finding, as low-income communities disproportionately experience high levels of community removal and social control via policing, arrests, incarceration, and surveillance [106,107]. Prior work has found that these can disrupt family networks and social relationships [108,109], and thus function as precursors to ACEs. As such, the association between TANF access and parental incarceration highlights the importance of economic resource provision to reduce the need for financially motivated offenses in the face of financial stressors [93]. However, further investigation can elucidate how to perform this resource provision more equitably so that TANF is well-situated to reduce parental incarceration irrespective of demographic differences.

It has been found that benefit reductions and bureaucratic hurdles to accessing welfare may contribute to adverse health consequences and exacerbate inequities [5,79,80,82,110,111]. Indeed, these limitations may in part explain the lack of a detectable relationship between TANF and other individual ACE outcomes in the present study, as well as the unexpected relationship between SNAP access and exposure to neighborhood violence and parental substance use [5].

One explanation for the unexpected associations observed between SNAP access and increased exposure to neighborhood violence and parental substance use may be the way we operationalized our exposure variable for SNAP access. Prior research documenting protective effects for families has generally evaluated state-level policy generosity or expansions (e.g., categorical eligibility, asset test removal, transitional benefits, removal of the drug felony ban), finding associations with reductions in child protective services involvement and foster care entries [31], lower rates of interpersonal violence [32], and declines in substance use disorders and unmet treatment need [112]. In contrast, our measure captures a state-level ratio of SNAP participation to individuals in poverty, reflecting program reach relative to need. This metric may be confounded by unmeasured structural factors, such as administrative capacity or burden (e.g., stricter rules suppress enrollment, while streamlined operations expand caseloads) [17,113], concentrated disadvantage (e.g., neighborhoods with high poverty density correlate both with SNAP participation and elevated risks of violence and substance use) [114,115,116,117,118], and other macroeconomic stressors (beyond unemployment spikes that drive up SNAP participation) [61] that are not fully accounted for in available covariates. Although our models adjust for certain state- and child-level factors, residual confounding from unobserved contextual variables likely persists, in part due to data availability constraints. Thus, a higher SNAP participation-to-poverty ratio may act as a proxy for structural hardship rather than directly confer protection. These measurement and confounding issues may account for the divergence from prior findings documenting the effects of SNAP policy generosity, underscoring the importance of cautious interpretation. Rather than overinterpreting these results as evidence for program harm, there is a need to further investigate these ecological dynamics and explore how structural and administrative conditions relate to program reach, and, in turn, childhood adversity.

Additionally, the contrasting findings for our TANF and SNAP models suggest that cash and near-cash programs may operate under different mechanisms. As suggested through evidence on cash programs in other countries, the cash benefits of TANF may provide families with flexible resources that can be used to meet diverse needs (e.g., transportation, utilities) that are closely tied to instability at the household level [119,120,121,122,123], functioning as compensatory factors within the Ecological-Transactional Model [43,44]. SNAP’s near-cash design, in contrast, may provide essential protection against food insecurity, a core determinant of health, but its restriction to food purchases may narrow its ability to buffer against a wider set of financial strains associated with ACEs, particularly when neighborhood-level factors such as concentrated poverty and community violence are present. How families experience these programs may also explain these findings. Both TANF and SNAP are burdened by stigma and surveillance, often rooted in racialized narratives of dependence [19,124,125]. TANF has historically been the more stigmatized program, since its limited reach targets households in deepest poverty, and given its widespread association with the PRWORA, which emphasizes personal responsibility, workfare mandates, and punitive sanctions [20,126,127,128]. At the same time, TANF benefits include cash, which families can use more flexibly [122,123]. SNAP, in addition to being more specific to food purchases, may also seem more visible at points of sale [66,114], rendering program design and stigma as possible potentiating factors. These experiential differences might suggest that, despite TANF’s heavier stigma, its relatively flexible design can function as a compensatory factor within the Ecological-Transactional Model, helping buffer against some adversities. SNAP’s narrower scope, and broader but less tailored reach, and greater visibility of use, may not offset potentiating factors to the same extent, which could help explain the inconsistent associations observed. It is also possible that TANF demonstrates greater promise in reducing exposure to some ACEs because it is specifically designed to target households with children, whereas SNAP more broadly targets low-income households with members of all ages [17,129]. Beyond structural differences in program design and intended recipients, the overlap between SNAP and TANF access is limited: while 82% of TANF participants accessed SNAP, only 7% of SNAP participants received TANF cash benefits in fiscal year 2022 [130,131]. This may partially explain the differential successes of TANF and SNAP access in curtailing specific types of ACEs.

It is critical to note that SNAP access rates are relatively high among Black children (45% in 2021); while no such estimates exist for children involved in state TANF programs, a smaller share of TANF recipients are Black individuals (30% in 2022) [132], reflecting TANF’s narrower reach. These racialized participation patterns reflect broader structural inequities. Black families are disproportionately likely to experience deep poverty and food insecurity [115,133,134], which may increase their reliance on SNAP. At the same time, they are more likely to encounter punitive barriers and restrictive rules that limit access to TANF, contributing to lower representation in that program relative to need [19,135]. The lack of promising associations observed from SNAP access is therefore concerning, as it raises the possibility that Black youth, who already experience a disproportionate burden of ACEs due to structural racism [136,137], may not currently be equitably protected by participation in these programs alone. As such, further investigation is needed to improve the accessibility and delivery of these programs so that they can serve the needs of youth of color, as they are most likely to be susceptible to the consequences of childhood poverty, including ACEs [138].

The present study’s findings on SNAP access should also be interpreted with caution because they stand in contrast to existing individual- and interpersonal-level studies conveying the protective association between food stamps and exposure to violence [32,139] and substance use [112]. This may suggest that additional contextual factors may be accounting for the relationships observed in this study. As this is the first study to examine welfare participation as a determinant of ACEs at the state level, additional research is needed to understand whether our results are replicable with other measures of welfare participation and child outcomes.

### 4.1. Policy Implications

Taken together, our results point to four domains of policy action to strengthen the primary prevention potential of TANF and SNAP: (1) reorienting TANF to function more fully as a family-centered income support, (2) enhancing SNAP’s adequacy while reducing administrative gatekeeping, (3) improving coordination across welfare programs and while systematically measuring ACE-relevant outcomes, and (4) expanding cash and near-cash supports to improve flexibility and equity. The following paragraphs expand on each of these domains.

Our findings indicate that greater TANF access is protective against parental incarceration and parental mental illness, though these relationships attenuate once child-level covariates are introduced. This suggests that TANF has the potential to buffer children from key adversities but that its current design may not deliver support equitably across demographic groups. Policies that increase benefit adequacy, reduce punitive sanctions, and ensure cash assistance is accessible to more families in deep poverty could amplify these protective relationships. Such reforms would align TANF with its statutory goal of enabling children to be cared for at home [18], and could reduce parental stressors that cascade into ACEs.

We found unexpected associations between higher level SNAP participation-to-poverty ratios and increased exposure to neighborhood violence and parental substance use. These findings likely reflect measurement issues and residual confounding, but they nonetheless raise concern that SNAP’s protective potential is not being fully realized. Strengthening SNAP’s adequacy through higher benefit levels, while streamlining enrollment and reducing exclusions (such as restrictions tied to drug convictions or immigration status), could better position the program to mitigate food insecurity and its downstream consequences. Such steps would address both the material insufficiency and administrative burdens that may currently blunt SNAP’s role in preventing ACEs.

The mixed results for TANF and SNAP also suggest that fragmented delivery may limit their combined protective potential. TANF demonstrated promise in reducing certain ACEs, while SNAP showed unexpected or null associations, despite both programs targeting overlapping populations. Better coordination (e.g., through harmonized eligibility rules, integrated cross-program data systems), could improve families’ ability to access multiple supports simultaneously. At the same time, embedding ACE-relevant measures into program evaluation may allow policymakers to directly monitor whether welfare reforms are achieving their intended protective relationship on child well-being, much like how social needs assessments and ACE screenings are increasingly incorporated into primary care settings to identify and address upstream drivers of health [140,141,142].

Our results highlight important contrasts between TANF and SNAP that suggest cash and near-cash programs may operate under different mechanisms, with differential protective associations across populations. Given TANF’s narrower reach and SNAP’s less consistent associations with ACEs, expanding the role of flexible cash and near-cash supports (including refundable tax credits and integrated TANF-SNAP delivery) can offer a way to strengthen equity within the safety net. Because cash and near-cash benefits can be used to meet diverse needs, they may reduce the inequities created when families must navigate fragmented, categorical programs that exclude certain expenses or populations. For example, unrestricted transfers enable households of color (who are more likely to encounter administrative barriers and punitive sanctions) to exercise agency in addressing housing, childcare, or transportation costs that disproportionately shape their exposure to adversity. By loosening these restrictions and ensuring access across racial and socioeconomic groups, flexible income supports may directly counter the structural inequities that limit the potential of existing welfare programs. In this way, reforms that prioritize cash and near-cash mechanisms can reduce racialized gaps in access and may facilitate families’ capacity to buffer ACEs.

These recommendations can be juxtaposed with recent U.S. policy changes: expansions of 2021 Child Tax Credit reduced child poverty by almost 50%, bringing it to record lows, by providing streamlined payments to families with children without the onerous application processes that characterize TANF and SNAP [143]. Related research on TANF during the COVID-19 pandemic similarly found that when states temporarily loosened requirements by issuing emergency cash benefits to non-participants, waiving work requirements, pausing sanctions, and automatically recertifying benefits, recipients experienced improved mental and physical health [77]. Similarly, guaranteed income pilots provide further evidence that unconditional transfers can reduce child poverty without discouraging work, which stands in sharp contrast to conditional assistance programs that are costly to administer, stigmatizing, and often trap families in cycles of low-paying and precarious work [144,145,146]. These examples suggest that simplifying welfare access and removing punitive conditions may amplify the U.S. safety net’s protective potential. In contrast, ongoing federal austerity measures proposes deep cuts to SNAP and other means-tested supports, risking a reversal of protective gains [147,148,149]. Together, such developments underscore the urgency of preserving and expanding flexible income supports rather than retrenching them.

Finally, while these program-specific recommendations offer concrete opportunities to strengthen TANF and SNAP as primary prevention strategies, it is important to situate them within the broader structural determinants of poverty and adversity. Long-term poverty alleviation and reductions in ACEs will require addressing the deeper drivers of structural inequity (e.g., inadequate wages, erosion of labor protections, disinvestment in public goods, and racially discriminatory economic policies) that shape families’ reliance on the safety net in the first place. Meaningful prevention, therefore, depends not only on improving the accessibility and adequacy of TANF and SNAP, but also on advancing structural reforms that ensure stable income, housing, healthcare, and community supports. At the same time, this study represents the first comprehensive effort to examine ACE outcomes in relation to state-level access to both major welfare programs. More research is needed to replicate and refine these findings, ideally using alternative measures of welfare participation and data sources that can capture local variation. Several limitations of the present study are discussed in the following section, signaling the need for cautious interpretation and continued investigation.

### 4.2. Limitations

The results from our secondary data analysis should be interpreted in light of limitations to the available data. With repeated cross-sectional data, we lack longitudinal insight into how outcomes for specific individuals may change over time. While using fiscal-year program data and calendar-year NSCH data improves temporal ordering by aligning exposures prior to outcomes, the partial overlap between fiscal and calendars introduces a small degree of ambiguity; nonetheless, this lag structure still offers stronger temporal clarity than simultaneous measurement. Additionally, as the NSCH relies on caregivers’ reporting of ACEs, rather than child reports, the data may be susceptible to social desirability bias given the sensitive nature of the questions. Relatedly, given these caregiver reports, the NSCH does not include all potential ACEs—only topics deemed capable of being “validly reported by parents/caregivers” [150]. A further limitation concerns the modeling of ACE outcomes. ACE items reflect distinct and causally heterogenous experiences that vary in severity and potential mechanisms of intervention. Collapsing them into a single total score provides parsimony and comparability to cumulative risk research but obscures item-specific pathways through which TANF or SNAP may shape relationships. We therefore prioritized item-level models to identify specific points of possible intervention, and supplemented them with robustness checks using the combined count to assess the coherence and robustness of overall relationships. In our robustness checks, TANF access was associated with lower cumulative ACE burden while SNAP showed no significant association.

Additionally, because our exposure variables are measured at the state level, the analysis may obscure heterogeneity within states. For example, counties or neighborhoods with higher concentrations of poverty, differential administrative practices, or varying local policy could experience distinct relationships between SNAP/TANF access and ACEs that are not captured in state-aggregated measures. Furthermore, in this context, state-level welfare participation cannot be assumed to translate directly to the experiences of individual households or children. Finally, there may be unmeasured confounding variables that relate to access to SNAP/TANF that are not accounted for within our covariates or fully captured by our state and year fixed effects.

## 5. Conclusions

Despite these limitations, there are multiple strengths to the study. Given the ethical and logistical constraints of withholding welfare from families assigned to the control group in a randomized experiment, our fixed effects approach offers an alternative to a randomized control trial to evaluate the effectiveness of SNAP and TANF access among families living in poverty in reducing ACE incidence. The study design also promotes causal inference by including external state-level and time trends that may relate to changes in welfare access and the incidence of ACEs. In addition, as the NSCH is a complex survey, the use of survey weights has addressed many issues related to missing data or nonresponse bias. Most critically, this study breaks new ground in the ACE literature by examining economic support as a primary prevention strategy—a proposal that has garnered national attention in response to the growing burden of ACEs in recent years [15,151,152,153,154], but has remained underexplored in the literature.

Taken together, our findings underscore the importance of considering structural interventions—such as cash and near-cash welfare programs—as primary prevention strategies to reduce ACE exposure. While TANF participation shows promise in mitigating certain household adversities, the mixed results for SNAP suggest that broader contextual or programmatic factors may shape outcomes. Future research should investigate how to strengthen these safety net programs, address differential impacts by race and socioeconomic status, and ensure that economic policies are designed and implemented in ways that truly buffer children from adversity. Building on the present study, future work could examine local variation within states, as counties and neighborhoods often differ in administrative practices, outreach strategies, and program uptake in ways that may shape ACE exposure. Studies could also incorporate richer measures of policy design and implementation (e.g., benefit “adequacy” or administrative burden) to identify which program features most strongly relate to family well-being and childhood adversity. Additionally, while our study focuses on structural levers such as TANF and SNAP, the impact of these programs may be conditioned on the presence of complementary interventions at the community, family, or individual level. For example, home visiting programs, social-emotional learning curricula, and trauma-informed care in healthcare and educational systems have delivered promising results for ACE-exposed youth [45,46,47,48,49,50,51]. Examining how such supports intersect with broader income and food assistance programs could help clarify the conditions under which protective factors are strengthened, moving toward a more comprehensive prevention approach. Further, disaggregated analyses by race, ethnicity, and nativity can help assess how structural racism and immigration status interact with welfare access to produce unequal outcomes for children. Linking welfare participation data to longitudinal child health and developmental outcomes would provide stronger evidence of causal pathways. Mixed-methods approaches that integrate caregiver perspectives with quantitative analyses could also help clarify how families experience TANF and SNAP in practice, including how bureaucratic hurdles or stigma may mitigate their potential protective relationships. This would allow us to move beyond evaluating whether welfare programs reach children in poverty, and assessing whether they are designed and delivered in ways that equitably reduce adversity and promote long-term health and stability. By advancing this line of inquiry, we can better harness policy levers to interrupt pathways that lead from poverty to adverse childhood experiences, ultimately improving health equity across the life course.

## Figures and Tables

**Table 1:** Weighted Sample Characteristics (*n* = 42,591).

Variable	Estimate	95% CI
**Child-level**		
**Age (years), mean (SD)**	9.20 (0.05)	(9.09, 9.30)
**Male (%)**	51.8	(50.8, 52.8)
**Race (%)**		
White	62.7	(61.7, 63.7)
Black	16.7	(15.9, 17.4)
American Indian/Alaska Native	2.6	(2.2, 3.0)
Asian	3.7	(3.4, 4.1)
Native Hawaiian/Other Pacific Islander	3.0	(2.5, 3.4)
Other	5.3	(4.7, 5.9)
Multiracial	6.0	(5.6, 6.5)
**Hispanic Ethnicity (%)**	43.3	(42.2, 44.3)
**Adverse Childhood Experiences (ACEs) (mean, SD)**		
Hard to cover basics like food or housing (%)	25.4	(24.5, 26.3)
Parent divorced or separated (%)	30.4	(29.5, 31.3)
Parent died (%)	4.8	(4.4, 5.3)
Parent served time in jail (%)	12.0	(11.4, 12.6)
Saw/heard adults hit each other in home (%)	7.2	(6.7, 7.6)
Victim/witnessed neighborhood violence (%)	5.8	(5.3, 6.3)
Lived with mentally ill/suicidal/depressed person (%)	7.8	(7.3, 8.3)
Lived with someone w/alcohol or drug problem (%)	10.3	(9.7, 10.9)
**State-level**		
State unemployment rate (%), mean (SD)	5.11 (0.02)	(5.07, 5.15)
State minimum wage ($), mean (SD)	8.82 (0.02)	(8.78, 8.87)
NSLP participation (1000 s), mean (SD)	1273.33 (17.21)	(1239.59, 1307.07)
SBP participation (1000 s), mean (SD)	697.73 (9.96)	(678.20, 717.25)
State poverty rate (%), mean (SD)	12.05 (0.02)	(12.01, 12.09)
TANF participation rate (TPR), mean (SD)	8.59 (0.16)	(8.29, 8.90)
SNAP gap (per 100 pop. below FPL), mean (SD)	106.81 (0.21)	(106.39, 107.22)

Note: Estimates are weighted and 95% confidence intervals account for the NSCH complex survey design. Analytic sample sizes ranged from 40,418 to 41,620 across models due to missing data (~5% missingness).

**Table 2:** Associations between TANF/SNAP access and ACEs: Results from two-way fixed effects models.

SNAP			
Outcome	Crude B (95% CI)	Adjusted B (95% CI)	Fully Adjusted B (95% CI)
Hard to cover basics like food or housing	0.37 (−0.33, 1.07)	0.12 (−0.92, 1.16)	0.12 (−0.91, 1.15)
Parent or guardian divorced or separated	−0.12 (−0.83, 0.59)	0.26 (−0.81, 1.34)	0.14 (−0.90, 1.19)
Parent or guardian died	0.04 (−0.28, 0.36)	−0.09 (−0.53, 0.35)	−0.11 (−0.54, 0.33)
Parent or guardian served time in jail	0.06 (−0.41, 0.52)	0.32 (−0.41, 1.04)	0.28 (−0.43, 0.98)
Saw or heard parents/adults hit each other in home	0.10 (−0.29, 0.49)	0.15 (−0.44, 0.75)	0.12 (−0.47, 0.71)
Victim of/witnessed violence in neighborhood	0.63 (0.25, 1.01) **	0.99 (0.42, 1.57) ***	0.95 (0.39, 1.51) ***
Lived with anyone mentally ill, suicidal, or depressed	0.25 (−0.15, 0.64)	0.14 (−0.44, 0.73)	0.16 (−0.41, 0.74)
Lived with anyone with alcohol or drug problem	0.48 (0.04, 0.93) *	0.39 (−0.29, 1.07)	0.38 (−0.29, 1.05)
Treated/judged unfairly because of race or ethnicity	0.30 (−0.05, 0.66)	0.26 (−0.23, 0.75)	0.21 (−0.27, 0.69)
**TANF**			
**Outcome**	**Crude B (95% CI)**	**Adjusted B (95% CI)**	**Fully Adjusted B (95% CI)**
Hard to cover basics like food or housing	−4.28 (−11.27, 2.71)	−3.27 (−10.03, 3.49)	−2.97 (−9.74, 3.80)
Parent or guardian divorced or separated	−2.90 (−10.07, 4.27)	−3.41 (−10.34, 3.51)	−1.85 (−8.73, 5.03)
Parent or guardian died	−0.86 (−3.20, 1.47)	−2.00 (−4.86, 0.85)	−1.65 (−4.45, 1.16)
Parent or guardian served time in jail	−3.98 (−8.09, 0.14)	−4.44 (−8.84, −0.05) *	−3.90 (−8.20, 0.41)
Saw or heard parents/adults hit each other in home	−1.87 (−5.08, 1.34)	−3.18 (−6.65, 0.29)	−2.77 (−6.22, 0.68)
Victim of/witnessed violence in neighborhood	−2.42 (−5.97, 1.13)	−3.11 (−6.41, 0.19)	−2.55 (−5.81, 0.70)
Lived with anyone mentally ill, suicidal, or depressed	−3.20 (−6.17, −0.23) *	−5.67 (−9.09, −2.26) ***	−5.40 (−8.80, −2.00) **
Lived with anyone with alcohol or drug problem	0.33 (−3.74, 4.41)	−2.14 (−6.52, 2.24)	−1.66 (−6.00, 2.68)
Treated/judged unfairly because of race or ethnicity	−0.84 (−3.17, 1.48)	−0.96 (−3.70, 1.78)	−0.57 (−3.29, 2.15)

* *p* < 0.05, ** *p* < 0.01, *** *p* < 0.001. Notes: Crude models only account for state and year fixed effects. Adjusted models include state-level covariates. Fully adjusted models include state- and child-level covariates. All models are survey-weighted with NSCH design variables. Model specific sample sizes range from 40,418 to 41,620 due to item non-response (~5%); no imputation was performed. All coefficients represent percentage-point change.

## Data Availability

Data used in this study are publicly available. State-level TANF and SNAP participation rates, as well as other state-level covariates, were obtained from the University of Kentucky Center for Poverty Research (UKCPR) National Welfare Data (1980–2022), available at https://cpr.uky.edu/resources/national-welfare-data (accessed on 11 June 2024). Individual-level data on adverse childhood experiences (ACEs) and child demographics were obtained from the National Survey of Children’s Health (NSCH) public use files, available at https://www.childhealthdata.org/learn-about-the-nsch/NSCH (accessed on 11 June 2024).

## Abbreviations

The following abbreviations are used in this manuscript:

ACEs	Adverse Childhood Experiences
TANF	Temporary Assistance for Needy Families
SNAP	Supplemental Nutrition Assistance Program
NSCH	National Survey of Children’s Health
PRWORA	Personal Responsibility and Work Opportunity Reconciliation Act
